# Efficacy and safety of Cerebrolysin in patients with hemorrhagic stroke

**Published:** 2010-05-25

**Authors:** O Bajenaru, C Tiu, H Moessler, F Antochi, D Muresanu, BO Popescu, P Novak

**Affiliations:** * Department of Neurology, University Hospital BucharestRomania; ** EBEWE Pharma, Unterach am AtterseeAustria; *** Department of Neurology, ‘Iuliu Hatieganu’ University of Medicine and Pharmacy Cluj NapocaRomania

**Keywords:** hemorrhagic stroke, Cerebrolysin

## Abstract

The purpose of the study was to investigate the efficacy and safety of Cerebrolysin in patients with hemorrhagic stroke. The primary objective of this trial was to assess the clinical efficacy and safety of a 10–days course of therapy with a daily administration of Cerebrolysin (50 mL Ⅳ per day). The trial had to demonstrate that Cerebrolysin treatment is safe in hemorrhagic stroke.

**Methods:** The study was performed as a prospective, randomized, double blind, placebo–controlled, parallel group study with 2 treatment groups. Efficacy measures were the Unified Neurological Stroke Scale, Barthel Index, and Syndrome Short Test. The duration of the trial was of 21 days for each patient. Out of 100 randomized patients, a total of 96 (96%) completed the study.

**Results:** Overall, no statistically significant group effects were observed based on single average comparisons at the individual visits. It could be shown that the treatment of hemorrhagic stroke with Cerebrolysin is safe and well tolerated.

**Conclusion**: In the changes of UNSS, BI and SST from baseline to day 21, the group differences are not statistically significant; however, the use of Cerebrolysin in hemorrhagic stroke is safe and well tolerated and studies with a larger sample size may provide statistical evidence of Cerebrolysin's efficacy in patients with hemorrhagic stroke.

## Introduction

The stroke is a major cause of mortality and disability, being responsible for about five million deaths worldwide each year [1]. Epidemiological data indicate that only cardiac disorders and cancer are the most common causes of mortality in Europe. The dramatic impact of stroke, on individuals, families and healthcare resources, is heightened by the long–term and frequently debilitating nature of its effects on mental and physical function and by its strong tendency to recur. Thereby, hemorrhagic stroke accounts for approximately 13% of all stroke incidences. However, taking a closer look at the stroke epidemiology, rates of ischemic and hemorrhagic stroke are higher in eastern than in Western Europe, North America or Australia [2], [3]. The incidence of stroke, in particular hemorrhage, is especially high in eastern Asia. In this area of the world stroke is the leading cause of death. Approximately 27% of strokes were secondary to hemorrhage. In eastern Asia, the relative proportion of hemorrhagic stroke is higher than that in other parts of the world [4]. The mortality rate in patients with hemorrhagic stroke is approximately four times higher than in patients with ischemic stroke [5], [6]. Only 38% of hemorrhagic stroke patients survive beyond the first year [7]. The management of patients with hemorrhagic stroke is generally limited to the supportive care or evacuation of the hematoma, although the efficacy of the surgical removal is variable and controversial [8], [9], [10].

Therefore, the need for a safe and effective treatment for patients with hemorrhagic stroke is urgently needed. There has been much interest in drugs that potentially protect neurons from the effects of ischemia, like NMDA receptor antagonists, antibodies to adhesion molecules, free radical scavengers, gangliosides, and apoptosis inhibitors. Encouraging results derive from studies in cell culture and *in vivo* stroke models after treatment with Cerebrolysin. The pre–clinical profile and the results of the previous clinical trials, provide the rationale for the assessment of the effects of Cerebrolysin treatment in patients with acute ischemic stroke and acute cerebral hemorrhage.

Cerebrolysin is a brain derived peptide preparation produced by a standardized enzymatic breakdown of lipid–free brain proteins and consists of low molecular weight peptides and free amino acids. It has been used in a number of European and Asian countries for various indications, for many years, having only rare and benign side effects reported. Cerebrolysin shows promise as a treatment for patients with an acute ischemic stroke. The effects of Cerebrolysin can be explained by its neuroprotective [11] and neurotrophic action [12], [13]. 

Data from earlier studies indicated a consistent trend in favor of Cerebrolysin as a treatment in patients suffering from an ischemic stroke [14], [15], [16], [17].

The first pilot trial indicated that Cerebrolysin could safely be used in cases of hemorrhagic stroke with a potentially beneficial clinical outcome [18].

The primary objective of this trial was to assess the safety and clinical efficacy of a 10–days course of therapy with a daily administration of Cerebrolysin (50 mL Ⅳ per day). 

## Method and Materials

### Study design

The design used in this study fulfilled all methodological requirements in order to prove the safety of a new treatment. The study was performed as a prospective, randomized, double blind, placebo–controlled, parallel group study with 2 treatment groups. In terms of ethical considerations, the treatment of all patients followed the guidelines regarding the standard treatment for cerebral hemorrhage. Four evaluation visits were scheduled for the enrolled patients. The patients were screened for study entry within 24 h from the onset of stroke. Eligible patients were then randomized to one of the two treatment groups and received the baseline evaluation of the efficacy outcome measures (visit 1, day 1). Immediately after the screening and baseline evaluation, patients received the first i.v. infusion of study medication. One group received 50 ml Cerebrolysin (n=51) the other group 50 ml placebo (n=49) for 10 consecutive days. Additional safety and clinical efficacy visits were scheduled on: day 4 (visit 2), day 10 (visit 3, end of active treatment) and on day 21 (visit 4).

The study was conducted at the University Hospital Bucharest, Department of Neurology. All patient data was collected at this single study center. The study was performed according to ICH–GCP guidelines and to the declaration of Helsinki and the protocol was approved by the national and local ethics committee of the study site. All patients and/or their relatives signed an informed consent form prior to the participation in the clinical study. The patients were recruited between December 2003 and April 2007.

### In– and exclusion criteria

The patient had to be between 40 and 85 years old (both inclusive) with a diagnosis of a first hemorrhagic stroke in the basal ganglia. The initial treatment with the trial treatment had to be administered between 6 and 24 hours after onset of symptoms.

Patients had to be excluded if a complete remission of symptoms occurred within the first few hours after stroke (i.e. patients who suffered from a TIA) or if the ischemic stroke or hemorrhagic stroke occurred in brain areas other than the basal ganglia, including the presence of the blood into the ventricles).

Furthermore, exclusion was necessary if the patient had uncontrollable hypertension (above 200/100 mmHg), an acute myocardial infarction, congestive heart failure, moderate to severe dementia prior to stroke, coma or stupor, accompanying severe diseases (cancer etc.), history of a stroke or other neurological disorders which impair evaluation. Other exclusion criteria were pregnancy or patients in lactation period, patients participating in other clinical trials, patients with chronic hepatic disorders or renal disorders/impaired renal function with serum creatinine above 1.5 mg/dl.

Patients were not included if he/she or his/her relatives refused to participate in the clinical trial.

### Patient population

**Figure 1:**
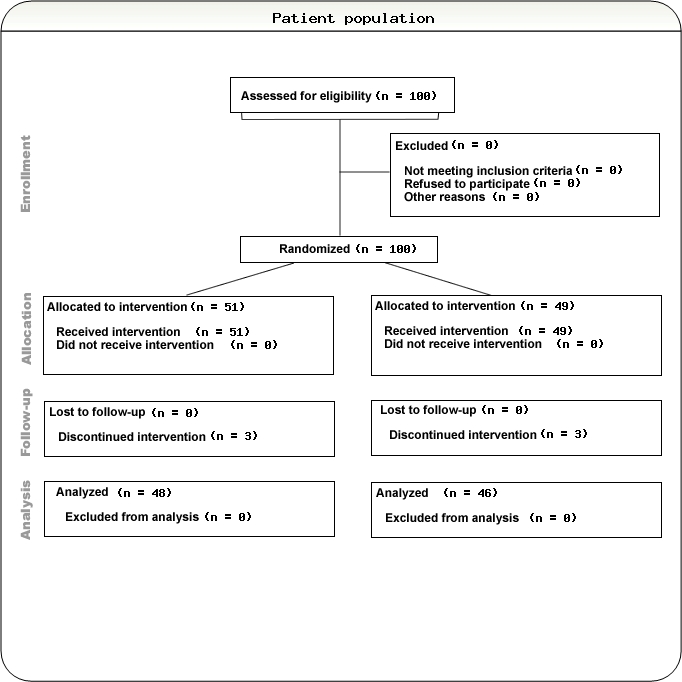


The trial was conducted in subjects suffering from first hemorrhagic stroke in the basal ganglia. The diagnosis of the hemorrhagic stroke was confirmed by Tomodensitometry (TDM). A total of 100 patients was randomized in the study and 96 patients (96%) completed the study. 49 patients received Placebo and 51 patients Cerebrolysin, out of which there were 62% male and 28% female patients with an average age of 65 years old. Efficacy measures were the Unified Neurological Stroke Scale, Barthel Index, and Syndrome Short Test. The duration of this trial for each patient was of 21 days. On inclusion day (visit 1) each subject received a baseline evaluation. The assessments and TDM were repeated on days 10 and 21. [Figure 1]

### Treatment and Randomization

Cerebrolysin 50 mL or placebo were administered once daily for 10 days by Ⅳ infusion in a peripheral vein. 

The study medication was provided by EBEWE Pharma and was packaged in amber glass vials containing 100 mL ready–to–use infusion solutions containing 50 mL Cerebrolysin and 50 mL normal saline (0.9% NaCl). The placebo infusion consisted of 100 mL of normal saline (0.9 NaCl). Both, active and control medication was packaged in 100 mL amber glass vials and were indistinguishable in appearance. Patients who met all inclusion criteria were assigned to one of the treatment groups in a 1:1 ratio according to a randomization code generated by computer software (EBEWE Pharma, Unterach, Austria). The investigators and all the study personnel were blind as to the random code assignment until the statistical analysis was finalized. A sealed envelope with information on the actual treatment for each patient was provided to the investigators for emergency cases. 

The dosage of Cerebrolysin in this study was chosen according to the clinical experience and was similar to the range of dosages used in previous clinical trials [19], [20].

### Safety evaluation

Adverse events (AE) and laboratory tests (blood chemistry, hematology and urinalysis) were evaluated for emergence of abnormal laboratory findings or changes in clinical laboratory tests and seriousness. Furthermore, changes in vital signs and general physical and neurological examinations were evaluated to determine the safety of Cerebrolysin. AEs were rated by the investigator as mild, moderate or severe. The relationship to the study drug was also rated by the investigator. The rating was done under blinded conditions. The patient's vital signs were measured at all visits, and a record of any adverse events was made. Additionally, an ECG was performed. A TDM was performed at visit 1 and 3 in order to determine the infarct volume. 

### Statistical analysis

An exploratory approach to the efficacy data was also used. Comparisons between groups at baseline were performed to warrant validation of proper randomization. This is defined as not exceeding a nominal two–sided 10% level of statistical significance. Kruskal Wallis H–tests followed at each visit, in case of statistical significance at the nominal two–sided 5% level by Wilcoxon Mann Whitney U–tests. At the same level, there is an appropriate method for an acceptable balance between rigid confirmatory objectives and power and sensitivity considerations in the context of the available sample sizes. Analysis of a one-way analysis of variance as a control was presented as an additional validation tool. This validation tool is essential to warrant a satisfactory quality control of the H–test and U–test methodology, based on discrete data as all those clinical scores are. 

The comparison between the two groups regarding the background and demographic characteristics was assessed by the use of descriptive statistics and appropriate parametric and non–parametric tests. Since this was an exploratory study, no calculation for the sample size has been performed. The sample size has been chosen according to experiences of previous clinical studies using Cerebrolysin.

All statistical analyses are based on the observed data set of the cases, and no imputation (e.g. last observation carried forward etc.) was performed. The statistical analysis was carried out by Neumann and Team, Vienna, Austria, by using a proprietary statistical software. This software has been tested and validated according to current guidelines. 

### Efficacy measurement methods

Changes from baseline (visit 1) to day 21 (visit 4) were used to assess the efficacy on the Unified Neurological Stroke Scale (UNSS), Barthel Index (BI), and Syndrome Short Test (SST). Efficacy measurements were performed at visits 1, 2, 3, and 4.

Visit 2 was performed on day 4 after Baseline. Visit 3 was performed at the end of the treatment on day 10, visit 4 on day 21. 

## Results

### Patient disposition

100 patients were randomized to the two treatment groups. 51 were allocated to the Cerebrolysin group and 49 to the control group. These 100 patients constituted the ITT population. Of these patients, 96 completed the study, 48 in the Cerebrolysin group and 46 in the control group. The reasons for discontinuation were 1 adverse event (skin rash) in the placebo group, 2 cases of death in each group and 1 case of consent withdrawal in the Cerebrolysin group. There were no obvious differences between the treatment groups with regard to the reason of study termination.

### Demographic data and baseline characteristics

Baseline demographic of the patients is presented in Table 1. No significant group differences of the demographic characteristics were observed at baseline. Heterogeneities at baseline variables are presented in Table 2 and a comparison of the efficacy parameters at baseline is given in Table 3. Patients in the Placebo group had a slightly better result in the Barthel Index at baseline.

**Table 1:** Demographic data Disorders

		Cerebrolysin N=51	Placebo N=49
Gender			
	Male	31	31
	Female	20	18
Age (years)		65	65
Height (cm)		168	168
Weight (kg)		73	76

The following variables displayed some statistically significant heterogeneities at the nominal 5% level at the baseline visit: 

**Table 2:** Heterogeneities at baseline variables

	Cerebrolysin	Placebo
variable	N=51	N=49
number of daily smoked cigarettes, mean	23	11
number of alcohol units/week, mean	11	7
heart rate, mean	80	76
respirations/min, mean	20	18
% abnormal pulmonary med. history	0%	10%
% history of alcohol, drug abuse	59%	22%
RBC, mean	4,7	4,9
% neutrophils, mean	78%	72%
Bicarbonate HCO_3_, mean	24	25
severity of first adverse event, % mild	24	25

Heart rate and respiration, as well as some other baseline variables shown in Table 2 were significantly different in the two groups at baseline. Diagnostic parameters as well as the severity of the disease were comparable across treatment groups. Furthermore, no relevant group differences were observed in demographic parameters, laboratory assessments, physical examinations and medical history of the patients.

**Table 3:** Efficacy parameters at baseline

	Cerebrolysin 50 mL N=51	Placebo N=49	Total N=100
SST results at Baseline, mean	21.725 ± 6.65	20.429 ± 7.40	21.090 ± 7.02
UNSS Index at Baseline, mean	38.725 ± 16.80	37.583 ± 21.06	38.172 ± 18.89
BI Index at Baseline, mean	23.431 ± 16.51	28.469 ± 21.07	25.900 ± 18.95

**Table 4:** Tomodensitometry data at baseline

	Cerebrolysin 50 mL N=49	Placebo N=42	Total N=91
Tomodensitometry at Baseline, mean	14.29 ± 11.76	11.61 ± 9.25	13.11 ± 10.80

From the statistical perspective, the two study therapy groups did not show any indications for some statistically significant group effects at the nominal 5% level of significance and, therefore, all the discrepancies which were observed, can be most likely attributed to the sampling error.

The SST results at baseline were comparable at baseline visit. The mean result for Cerebrolysin patients (n=51) was 21.725 ± 6.65 compared to 20.429 ± 7.40 for patients treated with placebo (n=49).

The UNSS results at baseline showed no significant differences at baseline as well. Patients treated with Cerebrolysin had a mean UNSS index of 38.725 ± 16.80 compared to 37.583  ± 21.06 in patients treated with placebo.

BI at baseline showed the following mean results: 23.431 ± 16.51 in patients treated with Cerebrolysin and 28.469  ± 21.07 in placebo treated patients. 

The tomodensitometry data, which were calculated in a post–hoc analysis, did not show any significant differences between the two groups at baseline, with 14,29 ± 11,76 cm^3^ in the active group and  11,61 ± 9,25 cm^3^ in the control group. 

### Efficacy

The efficacy was assessed by using the Short syndrome test, the Unified neurological stroke scale and the Barthel Index. 

Cognitive changes were assessed by using the SST. The change of SST from baseline to day 21 (visit 4) was 6.83 ± 6.71 and 4.39 ± 5.56 in the Cerebrolysin and placebo group, respectively. 

**Figure 2:**
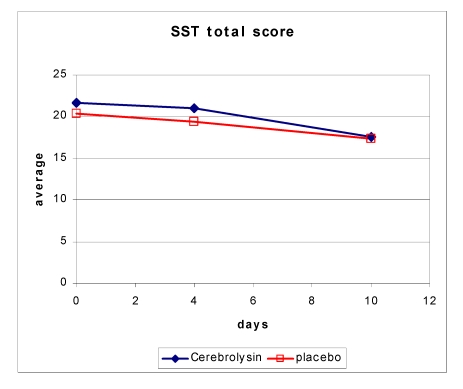
Change in SST total score over time

Neurological deficits were assessed by using the UNSS. The change in the UNSS total score from baseline to day 21 (visit 4) was 22.44 ± 12.83 in the Cerebrolysin and 23.98 ± 13.04 in the placebo group.

**Figure 3:**
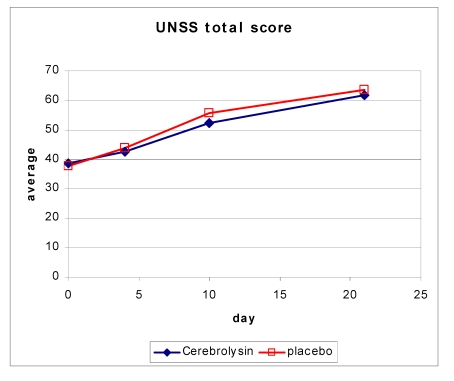
Change in UNSS score over time

The BI was used to assess the performance in basic activities of daily living. Over time, the BI score in the Cerebrolysin group changed by 27.81 ± 20.29 and in the control group by 29.67 ± 15.03.

**Figure 4:**
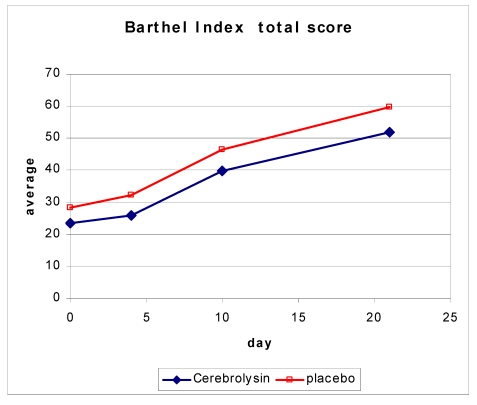
Change in BI score over time

Overall, there were no statistically significant group effects observed based on single average comparisons between the individual visits.

None of the in–between therapy group comparisons showed statistically significant group effects at the nominal 5% level, nor in the evaluation of changes also after a baseline adjusted analysis of covariance. 

In a post hoc analysis, the decrease in volume of the hemorrhage was calculated. 

**Table 5:** Change in volumetry of hemorrhage over time (in cm^3^)

	Day 1	Day 10	Day 21
Cerebrolysin	14.29 ± 11.76	10.92 ± 12.21	2.48 ± 4.62
Placebo	11.61 ± 9.25	9.32 ± 9.32	6.53 ± 6.53

**Figure 5:**
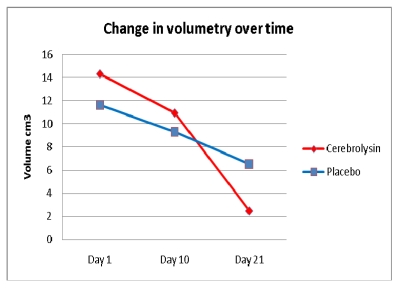
Change in volumetry of hemorrhage over time

Although the hemorrhage size was a little bit larger at day 1 in the Cerebrolysin group, the reduction in volume was slightly better on day 10 but distinctively better on day 21. A faster reduction in the hemorrhage size may contribute to a faster recovery in patients with hemorrhagic stroke.  

### Safety Results

The analysis of the Safety parameters showed that Cerebrolysin treatment with up to 50mL per day does not increase the incidence of the treatment's emergent adverse events as compared to the Placebo treatment. The analysis of the lab parameters, vital signs and ECG examinations did not provide any evidence for any substance specific toxic effect of Cerebrolysin. The treatment with Cerebrolysin with doses up to 50mL is safe and well tolerated.

The most common adverse event reported was urinary infection with 13 (25.5%) and 14 (28.6%) occurrences in the Cerebrolysin group and the placebo group, respectively. This was followed by increased body temperature with 10 (19.6%) and 8 (16.3%) occurrences in the Cerebrolysin and placebo group, respectively. Constipation occurred in 5 cases (9.8%) of the Cerebrolysin group and 9 cases (18.4%) of the placebo group. High blood pressure was reported in 6 (11.8) and 5 (10.2) patients of the Cerebrolysin and placebo group, respectively. Hypernatremia and Hypokalemia occurred in 6 (11.8%) patients of the Cerebrolysin group, but only 2 (4.1%) patients in the placebo group experienced Hypokalemia. There was no Hypernatremia reported in the Placebo group. All other individual events occurred less than 6 times across all study groups. Otherwise, there was no difference between the treatment groups as far as the incidence of the individual adverse events and the adverse events observed did not point to any substance related toxic effect of Cerebrolysin.

In total, 5 (5% out of 100) patients experienced at least one serious adverse event. This included 3 (3%) and 2 (2%) patients treated with placebo and Cerebrolysin, respectively. Overall, the incidence of SAEs was comparable in the two study groups. 

Only one patient (patient 144) discontinued the study due to AE (allergic skin rash). The investigational drug was stopped from being administered after the discontinuation of Ciprofloxacin did not improve the allergic reaction.

Among all cases, no patient receiving Cerebrolysin had severe adverse events, which were probably related to the treatment. One patient receiving Placebo experienced an allergic skin reaction, which was determined as possibly being related to the study drug. Because the allergic reaction did not stop after the discontinuation of Ciprofloxacin, the study drug was discontinued as well, and the event was considered to be possibly related to the study drug. 

For all other adverse events, the investigators assumed no causal relationship to the study medication. In accordance with this judgment, it can reasonably be assumed that none of the cases were caused by Cerebrolysin or Placebo. 

None of the events were repeatedly reported and they occurred with equal frequency in both treatment groups. There is no indication of any substance–specific effects of Cerebrolysin.

Overall, the number of SAEs was very low, given the indication of use in this study. None of the patients suffered a fatal SAE. The reason may be the fact that patients with very severe symptoms were excluded from the study. Therefore, the safety results were good in both groups and the primary endpoint, the safety of Cerebrolysin in hemorrhagic stroke could be proven.

## Discussion

In the changes of UNSS, BI and SST from baseline to day 21, the in–between group differences were not statistically significant; however, the use of Cerebrolysin in hemorrhagic stroke is safe and well tolerated and studies with a larger sample size may provide statistical evidence of Cerebrolysin efficacy in patients with hemorrhagic stroke.

Further dosage optimization could lead to further improvements of Cerebrolysin therapy.

Analysis of the Safety parameters showed that Cerebrolysin treatment with up to 50mL per day did not increase the incidence of Treatment Emergent Adverse Events as compared to Placebo treatment. The analysis of the lab parameters, vital signs and ECG examinations did not provide any evidence for any substance specific toxic effect of Cerebrolysin.

The treatment with Cerebrolysin with doses up to 50mL is safe and well tolerated.

The safety statement seems to be important for future, larger phase Ⅳ studies due to the fact, that this study data is the body of evidence that the safety profile of Cerebrolysin, under a homogenous set of concomitant medications and diseases is overall very well.

Further studies with a higher number of patients seem to have good chances to extend the findings of this trial and to experience positive results in the efficacy of Cerebrolysin in patients with hemorrhagic stroke. Therefore, patients with severe symptoms should be included in further studies to prove positive effects of Cerebsolysin.
